# 3 Tesla stack-of-stars echo unbalanced T1 relaxation-enhanced steady-state MRI for brain tumor imaging: post-contrast comparison with MPRAGE

**DOI:** 10.1186/s40644-025-00924-7

**Published:** 2025-08-15

**Authors:** Adrienn Tóth, Robert R. Edelman, Dmitrij Kravchenko, Justin A. Chetta, Jennifer Joyce, James Ira Griggers, Ruoxun Zi, Kai Tobias Block, M. Vittoria Spampinato, Akos Varga-Szemes

**Affiliations:** 1 Department of Radiology and Radiological Science, Medical University of South Carolina, 25 Courtenay Drive, Charleston, SC 29425 USA; 2 Medical Imaging Centre, Semmelweis University, Budapest, Hungary; 3 Department of Radiology, NorthShore University Health System, Evanston, IL USA; 4 Department of Radiology, Feinberg School of Medicine, Northwestern University, Chicago, IL USA; 5 The Bernard and Irene Schwartz Center for Biomedical Imaging, Department of Radiology, New York University Grossman School of Medicine, New York, NY USA

**Keywords:** Brain, Tumor, Metastases, 3T, MRI, Pulse sequence, Contrast

## Abstract

**Background:**

This study compared the image quality and diagnostic utility of stack-of-stars echo-unbalanced T1 relaxation-enhanced steady-state (SOS echo-uT1RESS) with the widely used magnetization-prepared rapid gradient-echo (MPRAGE) sequence in brain tumor imaging.

**Methods:**

In this prospective, two-center observational study, each participant underwent 3T contrast-enhanced MRI of the brain with both standard MPRAGE and prototype SOS echo-uT1RESS sequences. Lesion size, contrast-to-noise ratio (CNR), and tumor-to-brain contrast were quantitatively analyzed. Overall image quality, lesion conspicuity, and image artifacts were scored on a 4-point Likert scale, while diagnostic performance and assessment of the vascular and dural involvement were compared side-by-side by three readers.

**Results:**

Thirty-four adult patients (mean age, 64 years ± 13 [SD], 12 men) with known brain tumors (*N* = 6 intra-axial primary tumors; *N* = 14 intra-axial metastases; *N* = 14 extra-axial tumors) were enrolled in this study. There was no significant difference in CNR between MPRAGE and SOS echo-uT1RESS (29.4 ± 21.4 vs. 28.2 ± 16.5, respectively; *p* = 0.80, *r* = 0.03). SOS echo-uT1RESS demonstrated a 1.8-fold improvement in tumor-to-brain contrast compared with MPRAGE (0.7 ± 0.4 vs. 0.4 ± 0.3, respectively; *p* < 0.001, *r* = 0.81). While overall image quality and image artifacts were similar for both sequences, SOS echo-uT1RESS showed improved lesion conspicuity (*p* < 0.001, *r* = 0.51) and improved diagnostic performance (*p* < 0.001, *r* = 0.53), particularly for small metastases.

**Conclusion:**

SOS echo-uT1RESS enhanced lesion visibility, achieving approximately a 1.8-fold improvement in tumor-to-brain contrast compared to MPRAGE, although this finding may reflect both sequence properties and timing-related effects. The sequence maintained comparable overall image quality and robustness, making it a promising tool for brain tumor imaging.

**Supplementary Information:**

The online version contains supplementary material available at 10.1186/s40644-025-00924-7.

## Introduction

Contrast enhanced MRI plays a fundamental role in the diagnosis, anatomic delineation, and treatment planning of brain tumors [1–3]. Among established post-contrast sequences, magnetization-prepared rapid gradient-echo (MPRAGE) acquisition is widely utilized [4]. This technique enhances the anatomic brain tissue contrast between gray and white matter due to the inversion recovery preparation pulse, and it renders enhancing lesions and blood vessels conspicuously bright after gadolinium (Gd) contrast administration. However, the MPRAGE technique is limited in the visualization of small intracranial metastases and lesions with low Gd concentration [5, 6]. Detection of small brain metastases is of great clinical importance, as early diagnosis can expand treatment options such as stereotactic radiosurgery, improve prognosis, and potentially delay the onset of neurological symptoms [7].

Recent advances have introduced alternative MRI pulse sequences, which have demonstrated improved sensitivity for Gd-enhancing brain lesions [8–10]. Among such, the T1 relaxation-enhanced steady-state (T1RESS) pulse sequence has been shown to improve the visualization of brain tumors [11]. This pulse sequence selectively suppresses the signal intensity of non-enhancing background tissue while preserving that of Gd-enhancing lesions. Initial studies reported multiple advantages to the T1RESS pulse sequence, such as improved tumor-to-brain contrast, lesion sharpness, and a more consistent dark-blood effect compared to other neuroimaging techniques [11–13]. The dark-blood effect is especially pronounced using a “reversed fast imaging with steady-state free precession”-based (PSIF) version of the technique, called “echo-uT1RESS” [12]. Unfortunately, the same mechanism underlying the dark-blood effect can make the technique sensitive to head motion. Although post-acquisition motion correction techniques exist, they often fall shortly in fully restoring image quality in patients with significant or unpredictable movement– such as pediatric, elderly, or neurologically impaired patients [14]. Therefore, motion-robust acquisition strategies remain clinically important. To address this, a new version of the echo-uT1RESS technique was implemented using a motion-resistant radial stack-of-stars (SOS) k-space trajectory instead of the conventional Cartesian sampling [15, 16].

This study aimed to compare the image quality and diagnostic utility of the SOS echo-uT1RESS technique with the most commonly used post-contrast sequence, MPRAGE, in a patient cohort comprising both primary brain tumors and metastases.

## Materials and methods

### Patient cohort

This two-center prospective study received approval from the Institutional Review Board, and written informed consent was obtained from all participants. Eligible consecutive patients undergoing contrast-enhanced brain MRI were included between April 2023 and November 2024. Inclusion criteria were: standard-of-care imaging study within the prior year demonstrating at least one contrast-enhancing primary or secondary brain tumor; ability to tolerate the MRI procedure; and absence of contraindication to Gd-based contrast agent. Exclusion criteria were: safety-related contraindication to the MRI examination as determined by standard institutional guidelines and policies; inability to complete the informed consent form; estimated glomerular filtration rate < 30 ml/min/1.73 m² within the past 60 days; history of hypersensitivity to Gd-based contrast agents; and pregnancy or lactating.

### MRI acquisition protocol

All patients underwent native and contrast-enhanced brain MRI on 3T systems (MAGNETOM Skyra and MAGNETOM Skyra^Fit^, Siemens Healthineers, Erlangen, Germany). The complete imaging protocol included localizers, pre-contrast axial MPRAGE, axial T2 weighted imaging, fluid-attenuated inversion recovery, post-contrast MPRAGE, and SOS echo-uT1RESS acquisitions. Post-contrast imaging was performed after the intravenous administration of 0.1 mmol/kg gadobutrol (Gadavist, Bayer Healthcare, Malvern, PA, USA). Immediately after contrast administration, the 3D MPRAGE (T1-MPRAGE) acquisition was performed using the following typical pulse-sequence parameters: axial plane with TR 1900 ms, TE 2.45 ms, TI 900–1100 ms, flip angle 8–9°, 1.0 mm isotropic resolution, 250 Hz/pixel bandwidth, and a scan time of 4 min 52 s. Following MPRAGE, the SOS echo-uT1RESS dataset was acquired using the following parameters: axial plane with TR 6.9 ms, TE 4.16 ms, 1.0 mm isotropic resolution, 112 partitions, 512 radial views, sampling bandwidth 690 Hz/pixel, and a scan time of 6 min 51 s. The SOS uT1RESS sequence was acquired with a spatially nonselective saturation recovery preparation followed by a 3D reversed fast imaging with steady-state free precession (PSIF) readout. For each radial view, all 3D partitions are acquired as a single shot, followed by golden view angle rotation and acquisition of the next radial view [16]. To avoid gradient-induced spoiling of the steady-state signal when jumping from one radial view to the next, a flip-back gradient was applied along the radial-encoding direction after each readout. Detailed information on the image acquisition parameters is provided in Table 1. In one, randomly selected exemplary case, MPRAGE was acquired twice (before and immediately after SOS echo-uT1RESS) to control for potential timing differences in the observed contrast (Supplemental Figure S1). For the purpose of this study, only MPRAGE and SOS echo-uT1RESS images were evaluated. Multiplanar reconstruction was employed to generate sagittal and coronal views from the original images. These reconstructed images were then used for both quantitative measurements and qualitative assessments.

**Table 1:** Scan parameters for MPRAGE and SOS echo-uT1RESS

	MPRAGE	SOS echo-uT1RESS
Scan time	4 min 52 s	6 min 51 s
Signal averages	1	1
Voxel size (mm^3^)	1.0	1.0
Orientation	Axial	Axial
Field of view (mm)	256	288
Slices per slab	176	176
Shots per slice	1	1
Excitation	Selective	Nonselective
k-Space trajectory	3D Cartesian	Stack-of-Stars
Repetition time (ms)	1900	6.9
Echo time (ms)	2.45	4.16
Inversion time (ms)	900	1
Bandwidth (Hz/pixel)	250	690

MPRAGE = Magnetization-prepared rapid gradient echo; SOS echo-uT1RESS = Stack-of-stars echo unbalanced T1 relaxation-enhanced steady-state

### Quantitative analysis

Quantitative image analysis was performed by a radiology trainee (*A.T.*) using a picture archiving and communication system workstation (Sectra Medical, Sectra AB, Linkoping, Sweden). Region-of-interest measurements of signal intensity (SI) were obtained in the enhancing brain lesions, normal white matter (WM), and air. Due to limited gray-white matter contrast on SOS echo-uT1RESS images, initial ROI placement was performed on the MPRAGE series, where anatomical boundaries are more clearly defined. ROIs were then manually replicated on the SOS echo-uT1RESS images by aligning anatomical landmarks to ensure comparable positioning and size across sequences. Tumor regions-of-interest encompassed the entire enhancing lesion, excluding cystic and necrotic areas. Given that signal-to-noise ratio per voxel exceeded the Rose threshold of 4 [17], a metric analogous to Weber contrast served as the primary measure of lesion visibility [18], and was calculated as follows:

$$\:\text{W}\text{e}\text{b}\text{e}\text{r}\:\text{c}\text{o}\text{n}\text{t}\text{r}\text{a}\text{s}\text{t}=\frac{{\text{S}\text{I}}_{\text{t}\text{u}\text{m}\text{o}\text{r}}-{\text{S}\text{I}}_{\text{W}\text{M}}}{{\text{S}\text{I}}_{\text{W}\text{M}}}$$

where SI_tumor_ and SI_WM_ are the SIs measured in the enhancing lesion and the normal WM, respectively. All mention of “tumor-to-brain contrast” refer to the Weber contrast.

Additionally, contrast-to-noise ratio (CNR) was employed as a secondary metric, calculated as follows [19]:

$$\:\text{C}\text{N}\text{R}=\:\frac{{\text{S}\text{I}}_{\text{t}\text{u}\text{m}\text{o}\text{r}}-{\text{S}\text{I}}_{\text{W}\text{M}}}{{\text{S}\text{D}}_{\text{a}\text{i}\text{r}}}\times\:0.6555$$

where SI_tumor_ and SI_WM_ are the SIs measured in the enhancing lesion and the normal WM, respectively, while SD_air_ is the standard deviation (SD) of the air adjacent to the neurocranium.

For each lesion, the area (mm²) and diameter (mm) were recorded on axial-plane images from the slice where the lesion appeared largest.

### Qualitative analysis

MPRAGE and SOS echo-uT1RESS images were presented in a random order to three fellowship-trained neuroradiologist (*J.A.C.* with 15; *J.J.* with 10; and *D.K.* with 5 years of experience in interpreting brain MRI studies). The readers were blinded to clinical and demographic information and were unaware of the quantitative results. The evaluations were conducted using a picture archiving and communication system workstation (Sectra Medical, Sectra AB, Linkoping, Sweden). First, all readers assessed MPRAGE and SOS echo-uT1RESS images independently. Source images and multiplanar reformations were used to evaluate overall image quality; tumor conspicuity; and the presence of image artifacts. These assessments were scored on a 4-point Likert scale (Supplemental Tables S1-S3). Second, a side-by-side comparison was performed to directly assess the diagnostic performance, and the presence of dural or vascular invasion (Supplemental Tables S4-S5). Consensus reading was not employed in this study.

### Statistical analysis

Patient and examination characteristics were summarized descriptively. Normality of distribution was assessed using histograms and the Shapiro-Wilk test. The two-tailed Wilcoxon signed-rank test was used to assess differences in the distribution of qualitative and non-normally distributed quantitative image quality scores. One-tailed Wilcoxon signed-rank test was used to determine whether MPRAGE or SOS echo-uT1RESS improved the diagnostic performance and the detection of vascular or dural involvement. The inter-reader agreement of qualitative scores between all readers was quantified with Krippendorff’s alpha coefficients (α = 0.0–0.20, poor agreement; α = 0.21–0.40, fair agreement; α = 0.41–0.60, moderate agreement; α = 0.61–0.80, substantial agreement; α = 0.81–1.00, excellent (near perfect) agreement ). A p-value of less than 0.05 was considered statistically significant. If not stated otherwise, all data are presented as mean ± SD. All statistical analyses were performed using IBM SPSS, version 28.0.1.

## Results

### Patient population

**Fig. 1:**
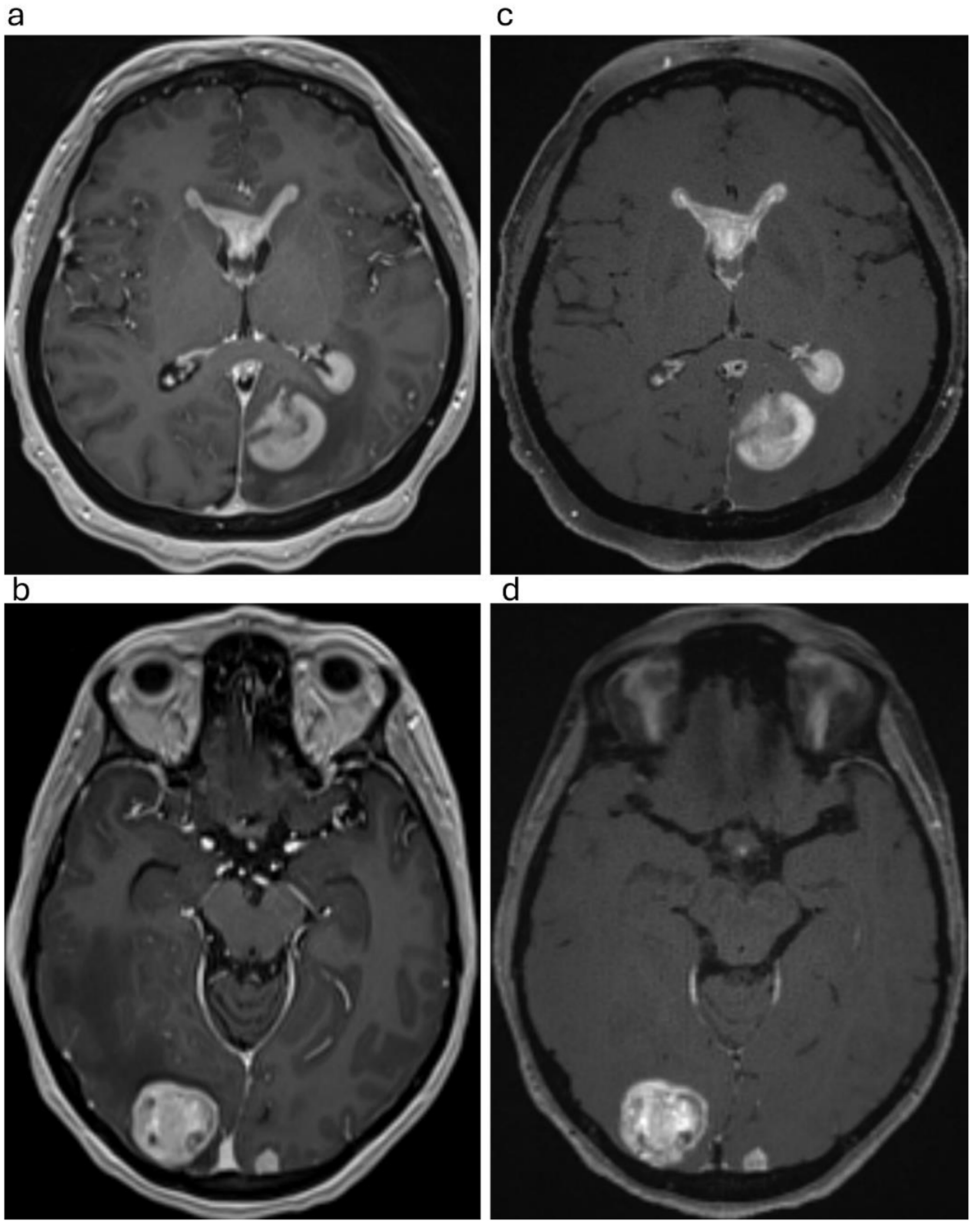
46-year-old patient with B-cell lymphoma (**a**,** c**) and 43-year-old patient with metastatic sarcoma (**b**,** d**). The comparison of axial plane images highlights the key differences between the two MRI pulse sequences: MPRAGE (**a**, **b**) and SOS echo-uT1RESS (**c**, **d**). MPRAGE provides excellent grey-white matter differentiation and clear delineation of peri-lesional changes, while the “background” brain tissue in SOS echo-uT1RESS appears more uniform. Additionally, SOS echo-uT1RESS provides dark blood features

**Fig. 2:**
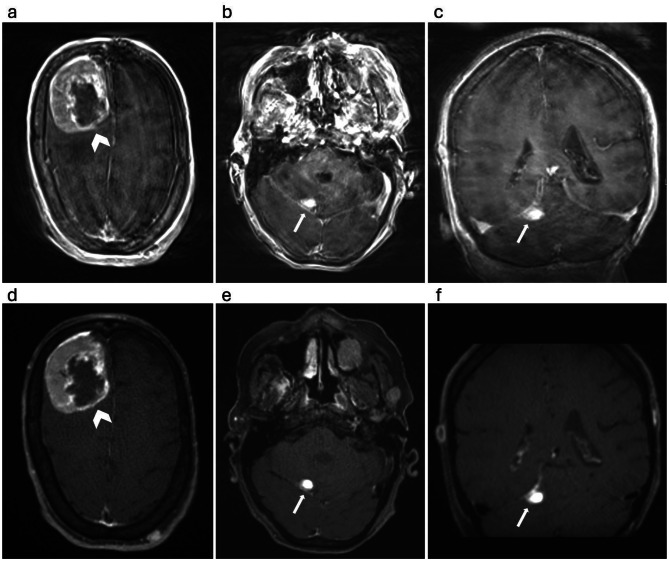
Patient with a large necrotic tumor and a smaller metastatic lesion. Axial (**a**, **b**) and coronal (**c**) plane MPRAGE; axial (**d**, **e**) and coronal (**c**) plane SOS echo-uT1RESS. The large necrotic tumor (arrow heads) and a smaller metastatic lesion (arrows) are well shown by both MPRAGE and SOS echo-uT1RESS sequences, however, the delineation of tumor margins is challenging in the presence of the significant motion artifacts observed on MPRAGE

**Fig. 3:**
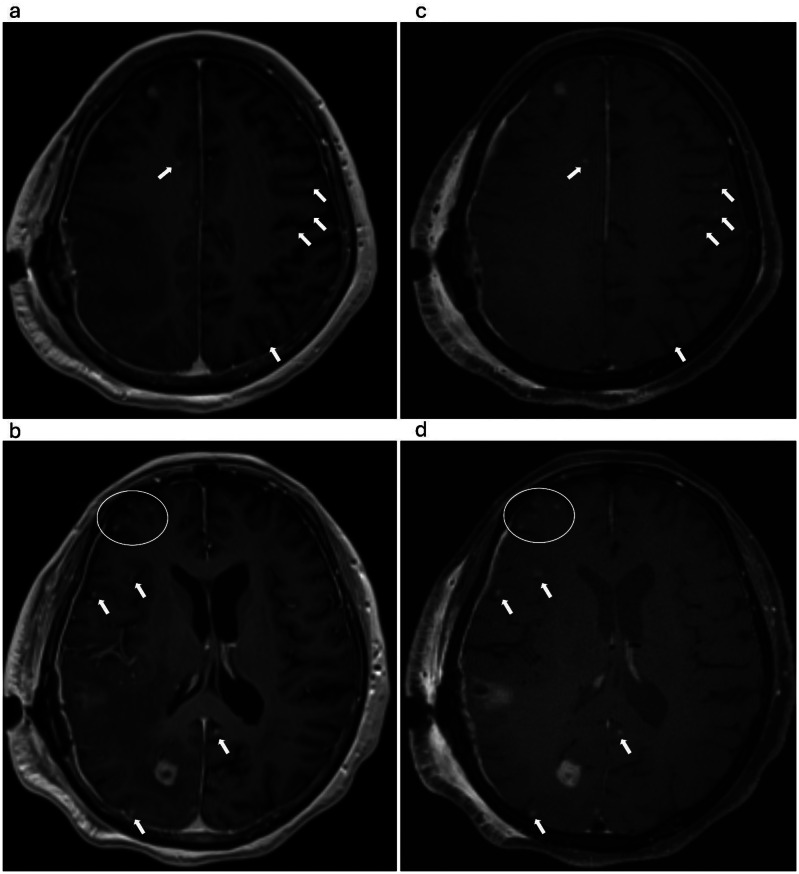
MPRAGE (**a**, **b**) and SOS echo-uT1RESS (**c**, **d**) acquisitions in a 76-year-old woman with metastatic lung cancer. The case is an example where, according to the readers, SOS echo-uT1RESS demonstrated superior diagnostic performance, with more than one lesion either better visualized or only apparent on this sequence (arrows and circles), but not on MPRAGE

**Fig. 4:**
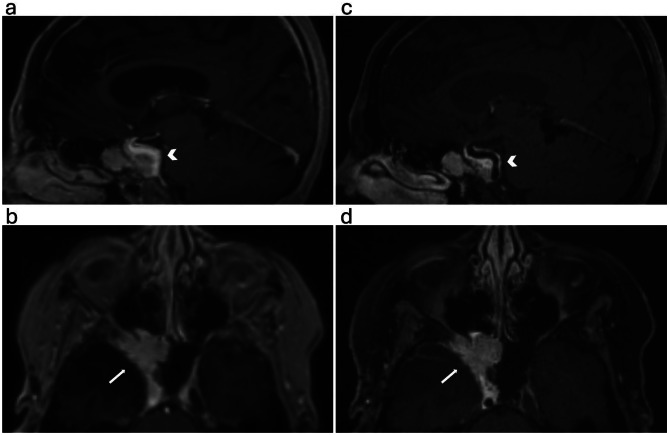
47-year-old woman with meningioma involving the right cavernous sinus and the Meckel’s cave (arrows). Sagittal (**a**) and axial (**b**) plane MPRAGE; sagittal (**c**) and axial (**d**) plane SOS echo-uT1RESS. The encasement of the right cavernous ICA with preserved flow void is well appreciated on the SOS echo-uT1RESS images (arrow heads)

Thirty-four adult patients diagnosed with a primary brain tumor or metastasis (22 women, age range 39 to 89 years) were imaged. Tumor types identified from biopsy or clinical history are provided in Table 2. Illustrative cases from the patient cohort are shown in Figs. 1, 2, 3 and 4.

**Table 2:** Patient demographics and tumor types

Characteristics	All patients (*N* = 34)
Man	12 (35%)
Woman	22 (65%)
Age, years (mean ± SD)	64.2 ± 13.4
Primary brain tumor	20 (59%)
Meningioma	14 (41%)
Ganglioglioma	1 (3%)
Glioblastoma	2 (6%)
Astrocytoma	1 (3%)
Lymphoma	2 (6%)
Metastatic disease	14 (41%)
Lung	6 (18%)
Breast	2 (6%)
Liver	2 (6%)
Renal	1 (3%)
Ovarian	1 (3%)
Sarcoma	1 (3%)
Melanoma	1 (3%)

N = number, SD = standard deviation

### Quantitative analysis

76 brain lesions in 34 patients were observed and measured. The average maximum lesion diameter and area on the reference MPRAGE images were 16.5 ± 10.0 mm and 197.1 ± 221.6 mm^2^, respectively. Measurements on SOS echo-uT1RESS images showed significantly larger lesion diameters (17.5 ± 10.4 mm, *p* < 0.001, *r* = 0.45) and areas (209.0 ± 223.8 mm^2^, *p* < 0.001, *r* = 0.50) compared to MPRAGE. There was no significant difference in CNR between MPRAGE and SOS echo-uT1RESS (29.4 ± 21.4 vs. 28.2 ± 16.5, respectively; *p* = 0.80, *r* = 0.03). Tumor-to-brain contrast across all patients and lesions showed significantly higher values with SOS echo-uT1RESS (0.7 ± 0.4) compared to MPRAGE (0.4 ± 0.3), representing an approximately 1.8-fold improvement with SOS echo-uT1RESS (*p* < 0.001, *r* = 0.81)(Table 3).

**Table 3:** Quantitative and qualitative image quality analysis

	MPRAGE	SOS echo-uT1RESS	*p*	*r*
Lesion size (mm)	16.5 ± 10.0	17.5 ± 10.4	**< 0.001**	0.45
Lesion area (mm^2^)	197.1 ± 221.6	209.0 ± 223.8	**< 0.001**	0.50
CNR	29.4 ± 21.4	28.2 ± 16.5	0.80	0.03
Tumor-to-brain contrast	0.4 ± 0.3	0.7 ± 0.4	**< 0.001**	0.81
Image quality	3.8 ± 0.5	3.8 ± 0.5	0.22	0.04
Lesion conspicuity	3.5 ± 0.9	3.8 ± 0.7	**< 0.001**	0.51
Image artifacts	3.7 ± 0.6	3.6 ± 0.6	0.30	0.06

MPRAGE = Magnetization-prepared rapid gradient echo; SOS echo-uT1RESS = Stack-of-stars echo unbalanced T1 relaxation-enhanced steady-state

### Qualitative analysis

**Fig. 5:**
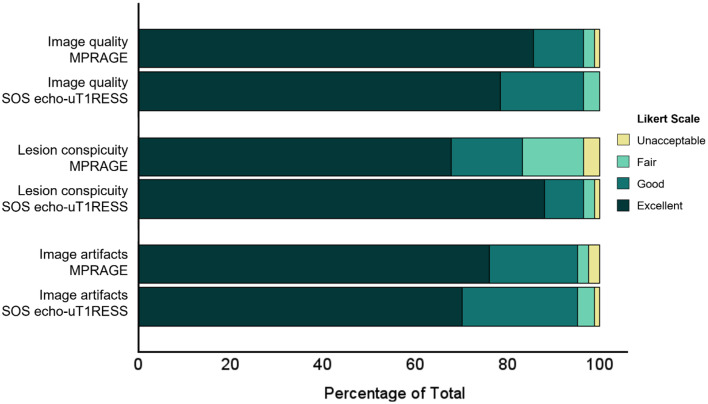
Stacked bar charts display the results of the qualitative image quality analysis. The images were rated by three independent readers based on overall image quality, lesion conspicuity and image artifacts using a 4-point Likert scale (1– unacceptable; 4– excellent)

Overall image quality was rated good or excellent in the majority of the cases and revealed no statistically significant difference between MPRAGE and SOS echo-uT1RESS sequences (3.8 ± 0.5 vs. 3.8 ± 0.5, respectively; *p* = 0.22, *r* = 0.04). Additionally, the presence of image artifacts was found to be similar with the two sequences (3.7 ± 0.6 vs. 3.6 ± 0.6, respectively; *p* = 0.30, *r* = 0.06). SOS echo-uT1RESS demonstrated significantly improved conspicuity of enhancing lesions (3.5 ± 0.9 for MPRAGE vs. 3.8 ± 0.7 for SOS echo-uT1RESS, *p* < 0.001, *r* = 0.51), consistent with the results of the quantitative tumor-to-brain contrast measurements (Table 3**and** Fig. 5).

**Fig. 6:**
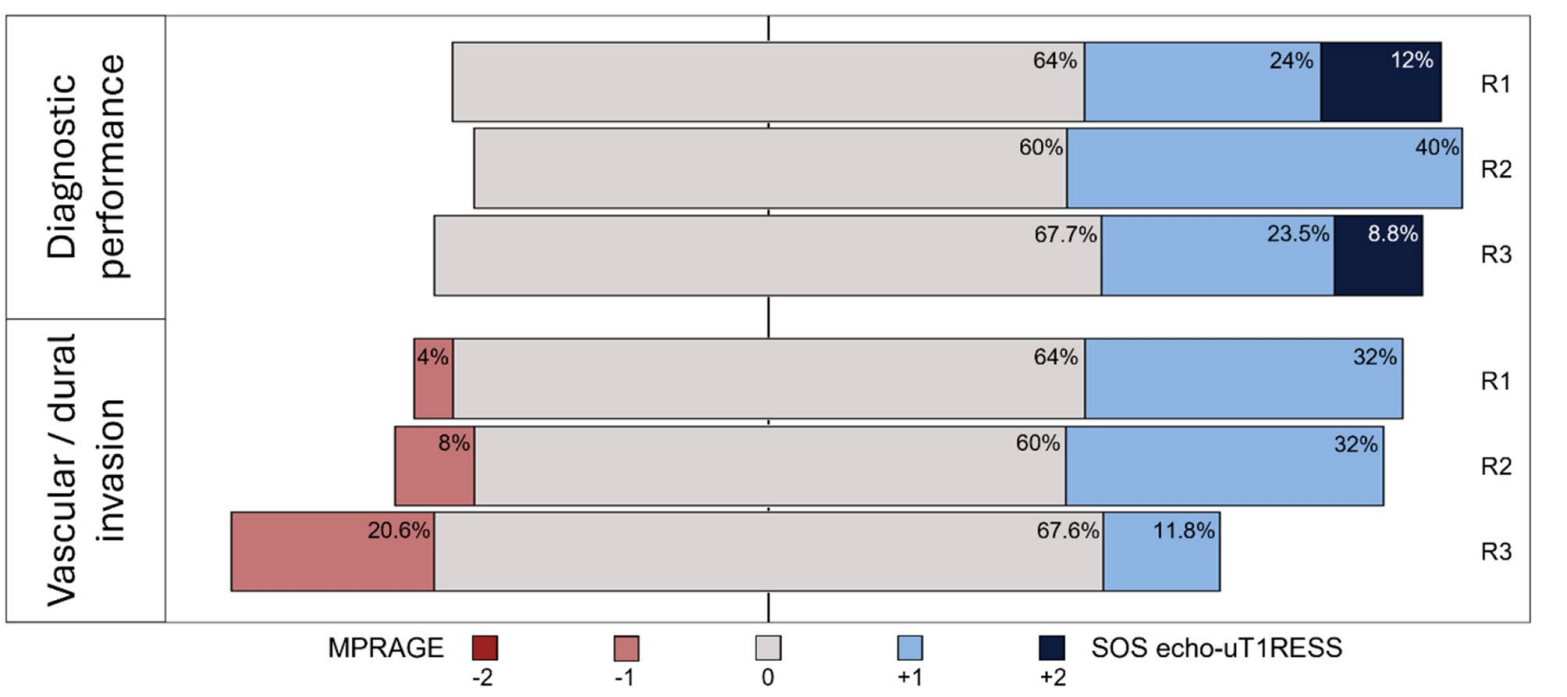
The diverging bar chart displays the results of the side-by-side qualitative image quality analysis performed by three readers (R1-R3). The images were rated using ordinal scales, e.g.: (-2) one or more enhancing lesions only shown by MPRAGE; (-1) one or more lesions better shown by MPRAGE; (0) lesions equally well shown by MPRAGE and SOS echo-uT1RESS; (+ 1) one or more enhancing lesions better shown by SOS echo-uT1RESS; (+ 2) one or more lesion only shown by SOS echo-uT1RESS

Diagnostic performance significantly improved with SOS echo-uT1RESS sequence (*p* < 0.001, *r* = 0.53) (Fig. 6). Specifically, Reader 1 rated SOS echo-uT1RESS as superior in detecting lesions in over 35% of the cases, with one or more lesions appearing exclusively on this sequence and not on MPRAGE in 12%. Reader 2 reported that SOS echo-uT1RESS improved the visibility of one or more lesions in 40% of the cases examined.

The majority of cases where SOS echo-uT1RESS enhanced diagnostic confidence involved brain metastases of various origins, typically characterized by lesion sizes smaller than 10 mm (Fig. 3). In general, detection of vascular or dural involvement showed no significant differences between SOS echo-uT1RESS and MPRAGE (*p* = 0.068, *r* = 0.16). Both Reader 1 and Reader 2 preferred SOS echo-uT1RESS over MPRAGE in 30% of the cases for evaluating the vascular or dural involvement. However, in a total of seven cases (comprising four metastases and three primary brain tumors), MPRAGE outperformed SOS echo-uT1RESS in depicting dural or vascular invasion, as small prominent vessels and vascular structures appeared more tumor-like on SOS echo-uT1RESS. Interobserver agreement for the qualitative image scores was substantial (α = 0.76).

## Discussion

This study demonstrated that the post-contrast SOS echo-uT1RESS technique significantly enhances quantitative tumor-to-brain contrast, thereby providing superior visual lesion conspicuity compared to the clinically used post-contrast MPRAGE sequence. Moreover, the technique maintains similarly high overall image quality to MPRAGE with comparable levels of image artifacts from patient motion or other sources. Given issues with motion sensitivity using a previously described Cartesian version of echo-uT1RESS [12], we ascribe the motion robustness of the current technique to the use of a radial stack-of-stars k-space trajectory. Small metastases and weakly enhancing lesions were notably more visible due to improved tumor-to-brain contrast with SOS echo-uT1RESS, enabling the detection of as small as 2–3 mm in diameter lesions initially not observed on MPRAGE.

Previous studies comparing different versions of the uT1RESS sequence with MPRAGE have consistently reported several advantages of the new technique, including enhanced quantitative tumor-to-brain contrast, improved visual conspicuity of lesions, and effective suppression of blood vessel and cerebrospinal fluid signal intensities, which were reaffirmed in this study [11–13]. In general, the T1RESS technique enhances tumor visibility in contrast-enhanced MRI by selectively suppressing the SI of non-enhancing background tissues while preserving the signal intensity of contrast-enhancing lesions. Unlike MPRAGE, which uses radiofrequency spoiling, T1RESS employs gradient spoiling, fundamentally altering contrast behavior. MPRAGE renders both enhancing lesions and blood vessels bright post-Gd administration. However, for certain pathology evaluations, dark-blood imaging can offer advantages. The previously described echo-uT1RESS version of this technique renders blood vessels dark using a steady-state readout with unbalanced gradients [13].

While SOS echo-uT1RESS requires a longer acquisition time than MPRAGE, it allows for the application of GRAPPA (GeneRalized Autocalibrating Partially Parallel Acquisition) in the *k*_*z*_ direction. This feature can help reduce the overall scan time by accelerating the imaging process without compromising image quality, thereby making SOS echo-uT1RESS a valuable option for improving diagnostic capabilities despite its increased acquisition time [20]. In our implementation, SOS echo-uT1RESS required approximately 30% longer table time than MPRAGE. Although the longer acquisition time represents a clear limitation in time-sensitive clinical workflows, it is partially offset by the sequence’s improved lesion conspicuity and inherent motion robustness. The radial Stack-of-Stars trajectory is less sensitive to patient movement compared to traditional Cartesian techniques and may result in fewer nondiagnostic exams in real-world settings, particularly among pediatric, elderly, or uncooperative patients. Future work should investigate the diagnostic yield and image quality of faster SOS echo-uT1RESS variants using higher acceleration factors or compressed sensing to reduce scan time while preserving image quality.

Our results revealed that measurements using SOS echo-uT1RESS demonstrated slightly larger lesion sizes and areas than those observed with MPRAGE. This difference may reflect the increased sensitivity of SOS echo-uT1rESS to subtle peripheral gadolinium enhancement, resulting in more conspicuous lesion margins and slightly expanded measured tumor dimensions. Danieli et al. recently conducted a morphometric assessment of enhancing brain tumors comparing MPRAGE, VIBE (volumetric interpolated brain examination), and SPACE (sampling perfection with application-optimized contrast using different flip angle evolution) MRI techniques. They reported morphometric discrepancies in brain tumor segmentation, noting significantly larger tumor volume estimates with SPACE compared to MPRAGE [9]. As our study was not designed for detailed morphometric measurements, only lesion size and area were assessed, however, this aspect warrants consideration in future studies. Additionally, in this study, MPRAGE served as the reference technique as the standard clinical practice. Future studies should therefore explore comparisons between SOS echo-uT1RESS with these alternative techniques.

This study has several limitations. First, SOS echo-uT1RESS is a prototype pulse sequence that is fundamentally different from existing neuroimaging techniques. As such, further optimization and pre-clinical testing are necessary before the sequence can be fully refined for clinical application. Second, while advantageous for detecting enhancing lesions, SOS echo-uT1RESS images do not provide sufficient detail for gray/white matter segmentation due to their bland appearance of normal brain tissue. Moreover, in some cases, MPRAGE outperformed SOS echo-uT1RESS in depicting dural or vascular invasion, as incomplete suppression of small vessels on SOS echo-uT1RESS caused these structures to appear more tumor-like, potentially leading to false-positive interpretations. A recent study by Lasocki et al. reported a similar phenomenon, where T1 SPACE improved detection of small melanoma metastases but also introduced an increased false-positive burden attributed to vascular artifacts, as correlated with MPRAGE [21]. In contrast, over-suppression of vascular structures may reduce the visibility of small enhancing lesions located adjacent to vessels, leading to false-negative findings, as demonstrated in a recent study by Fu et al., where 12 small brain metastases were initially missed on the vessel-suppressed DANTE-SPACE acquisition and misinterpreted as normal enhancing vessels at the brain surface [22]. Therefore, the inclusion of additional sequences acquired before or after the SOS echo-uT1RESS acquisition remains necessary to ensure accurate interpretation in clinical practice. Third, in the absence of biopsy confirmation, not all enhancing lesions identified may represent actual tumors. Long-term patient follow-up studies are required to ascertain the nature and clinical significance of these lesions. Additionally, while several comparisons reached statistical significance, it is important to note that some of the observed differences - such as those in lesion size and conspicuity - were relatively small in absolute magnitude. Future studies with larger sample sizes and outcome-based validation are warranted to better assess the clinical relevance of these differences. Finally, it was not possible to randomize the sequence order within the current study design, as it was necessary for clinical purposes to obtain all standard neuroimaging sequence prior to the test sequence. This introduces a potential bias due to progressive contrast accumulation over time. We sought to mitigate this by including one exemplary case in which MPRAGE was acquired both before and after SOS echo-uT1RESS, which demonstrated contrast improvement between the first and second MPRAGE acquisitions. Although timing slightly improved tumor-to-brain contrast between the first and second MPRAGE scans, CNR remained comparable. Moreover, SOS echo-uT1RESS yielded the highest CNR and tumor-to-brain contrast values among the three acquisitions. This finding highlights the influence of contrast timing on lesion conspicuity; however, the observed superiority of SOS echo-uT1RESS likely reflects a combined effect of delayed enhancement and intrinsic sequence properties. This interpretation is further supported by the fact that no consistent bias in sequence performance has been observed in prior published studies comparing unbalanced T1RESS and MPRAGE. Future studies with randomized acquisition order will be essential to better separate the effects of contrast timing from those of the sequence itself.

Relative to T1 SPACE, which is the primary technique currently used for post-contrast dark blood 3D imaging of brain tumors, potential benefits of the radial stack-of-stars echo-uT1RESS technique include greater resistance to motion artifacts, absence of blurring due to T2 decay during the echo train, and the capability to generate dark blood time-resolved perfusion images using a Golden-Angle Radial Sparse Parallel (GRASP) reconstruction technique [13, 23]. However, direct sequence comparisons will be needed in future studies to assess the relative advantages and drawbacks of each technique.

In conclusion, this study underscores the potential of the SOS echo-uT1RESS technique as an advanced MRI pulse sequence for improving the visualization and characterization of brain lesions. It demonstrated significantly enhanced quantitative tumor-to-brain contrast and superior lesion conspicuity compared to the conventional MPRAGE sequence, while maintaining high overall image quality with minimal artifacts. The technique’s capability to highlight small metastases and weakly enhancing lesions, not readily observable with MPRAGE, suggests promising clinical applications.

## Supplementary Information

Below is the link to the electronic supplementary material.

Supplementary Material 1

## Data Availability

The datasets used and/or analyzed during the current study are available from the corresponding author on reasonable request.

## Abbreviations

CNR: Contrast-to-noise ratio
echo-uT1RESS: Echo unbalanced T1 relaxation-enhanced steady-state
MPRAGE: Magnetization-prepared rapid gradient echo
SOS: Stack-of-stars
SPACE: Sampling perfection with application-optimized contrast using different flip angle evolution
VIBE: Volumetric interpolated brain examination
WM: White matter
