# Supplementary material for: 3 Tesla stack-of-stars echo unbalanced T1 relaxation-enhanced steady-state MRI for brain tumor imaging: post-contrast comparison with MPRAGE
Source: Cancer Imaging. 2025 Aug 15;25:101. doi: 10.1186/s40644-025-00924-7 (PMC12357450; doi:10.1186/s40644-025-00924-7)
Supplement: Supplementary file 1 — Supplementary Material 1 [file 40644_2025_924_MOESM1_ESM.docx]

**SUPPLEMENTAL MATERIAL**

**Supplemental Tables**

| Table S1 – 4-point Likert scale for the assessment of overall image quality | |
| --- | --- |
| Score | Interpretation |
| 1 | Unacceptable / nondiagnostic image quality |
| 2 | Fair / acceptable image quality |
| 3 | Good image quality allowing for confident diagnosis |
| 4 | Excellent image quality allowing for highly confident diagnosis |

| Table S2 – 4-point Likert scale for the assessment of lesion conspicuity | |
| --- | --- |
| Score | Interpretation |
| 1 | Barely noticeable lesion, lesion margins or internal structure are not assessable |
| 2 | Identifiable lesion, lesion margins or internal structure are distorted |
| 3 | Clearly visible lesion, lesion margins or internal structure are only mildly distorted |
| 4 | Distinctly visible lesion, excellent delineation of lesion margins and/or internal structure |

| Table S3 – 4-point Likert scale for the assessment of image artifacts | |
| --- | --- |
| Score | Interpretation |
| 1 | Severe artifacts |
| 2 | Moderate artifacts |
| 3 | Mild artifacts |
| 4 | No clinically relevant artifacts |

| Table S4 – 5-point scale for the side-by-side assessment of the diagnostic performance | |
| --- | --- |
| Score | Interpretation |
| -2 | One or more enhancing lesions only shown by MPRAGE |
| -1 | One or more enhancing lesions better shown by MPRAGE |
| 0 | Lesions equally well shown by MPRAGE and SOS echo-uT1RESS |
| +1 | One or more enhancing lesions better shown by SOS echo-uT1RESS |
| +2 | One or more enhancing lesions only shown by SOS echo-uT1RESS |

| Table S5 – 4-point scale for the side-by-side assessment of the presence of vascular and/or dural involvement* | |
| --- | --- |
| Score | Interpretation |
| -1 | Better shown by MPRAGE |
| 0 | Equally well shown by MPRAGE and SOS echo-uT1RESS |
| +1 | Better shown by SOS echo-uT1RESS |

* Vascular invasion was defined based on morphologic signs, including encasement, narrowing, or loss of normal vessel contour, as well as tumor signal within the expected location of vascular structures. Dural involvement was defined as nodular or linear contrast enhancement along the dura mater adjacent to the tumor, suggesting dural infiltration or spread.

**Supplemental Figures**


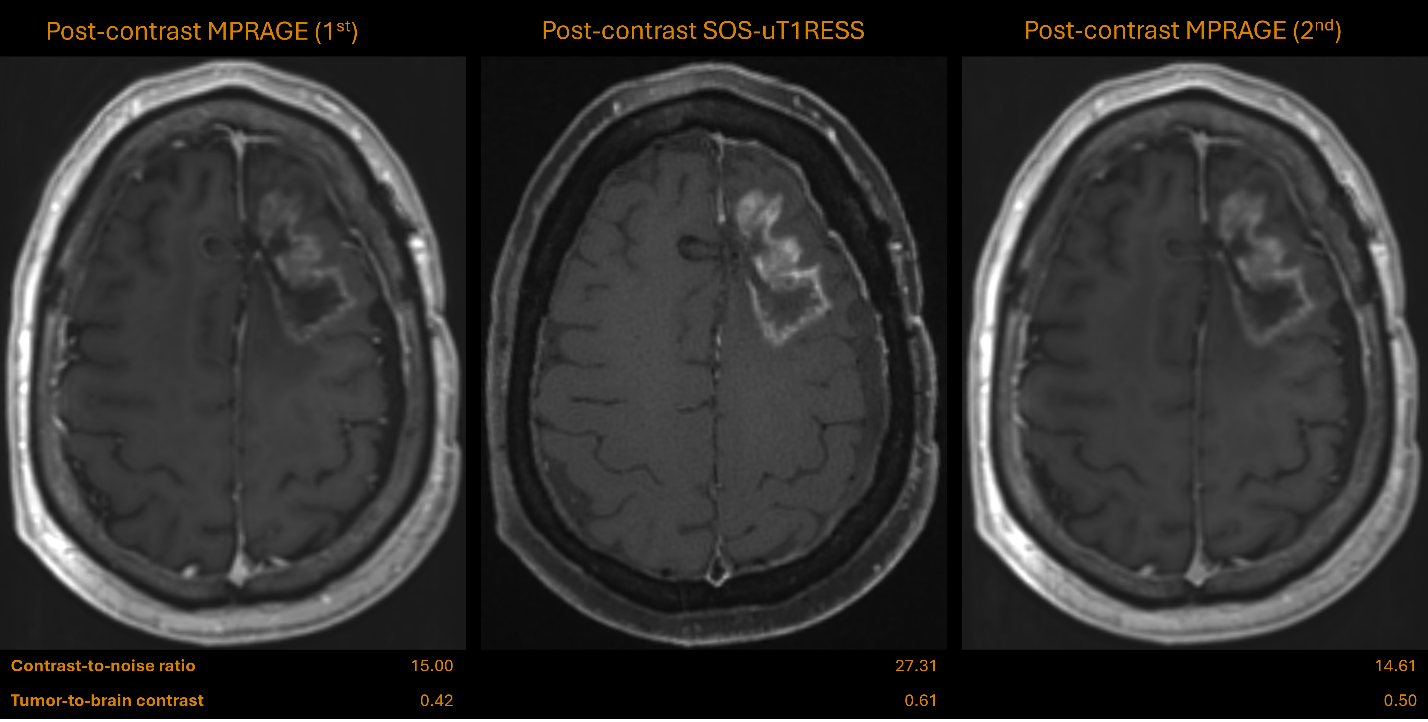


**Figure S1.** The figure presents three axial images along with the measured contrast-to-noise ratio (CNR) and tumor-to-brain contrast values from an exemplary case, illustrating the differences observed between the MPRAGE acquisitions and the SOS echo-uT1RESS sequence. While timing led to some improvement in tumor-to-brain contrast from the first to the second MPRAGE acquisitions, SOS echo-uT1RESS yielded the highest values of CNR and tumor-to-brain contrast among the three acquisitions.
